# Low light image enhancement using curvelet transform and iterative back projection

**DOI:** 10.1038/s41598-023-27838-3

**Published:** 2023-01-17

**Authors:** Sreekala Kannoth, Sateesh Kumar H. C., Raja K. B.

**Affiliations:** 1 Visvesvaraya Technological University, Belagavi, Karnataka India; 2 Department of ECE, Sapthagiri College of Engineering Bengaluru, Bangalore, Karnataka India; 3 Department of ECE, University Visvesvaraya College of Engineering, Bangalore, Karnataka India

**Keywords:** Engineering, Electrical and electronic engineering

## Abstract

With the advancement of technology in image capturing, people are accustomed to high-resolution images. One of the primary necessities of an image capturing system is to provide the same. However, in many cases, the image resolution may not be reaching the expectations of the user which leads to a decrease in user experience. This is a common phenomenon that occurs when the images are captured in low light or if the image encounters a distortion either because of lack of exposure or the image capturing devices may be equipped with a small size sensor. In this work, a resolution enhancement technique using the concepts of curvelet transform and iterative back projection is presented. Sparse representation of images can be enhanced using a combination of curvelet transforms with iterative back projection. Application of curvelet transform along with iterative back projection algorithm on low light images results in enhancing the resolution of the images. The resultant images from here then passed through the inverse transform block and gives an image with contrast enhancement which leads to the user experience improvement. The antiquated image enhancement with improvement in the resolution is validated with the measurement of peak signal-to-noise ratio and structural similarity index. The usage of curvelet transform with iterative back projection leads to the restoration of the image resolution by minimizing the distortions, thus leading to an enhanced image whose edge details are retained.

## Introduction

Resolution enhancement of images is a key requirement especially when the images are captured in an environment where the light present is of considerably low. Such images which we call low light images are often subjected to a lot of degradation in terms of noise and resolution and thus a mechanism to improve the same is of utmost concern. In such cases, as an addition to resolution enhancement, an improvement over the contrast by maintaining the edge details is considered to be a value addition to the image processing techniques to be applied. High resolution images find their applications spread over a number of varied areas but are not limited to the field of medical imaging, remote sensing, satellite imaging for the classification of urban land areas, and other commercial applications. When such images are combined with enhanced contrast, the tremendous potential of its applicability encompasses even wider regions. This creates the potential for this research work which concentrates on tapping the same into more meaningful image processing techniques.

With the tremendous advancements in technology especially related to image capturing and video capturing, there has been a multi-fold increase in the number of applications making use of the same. The image capturing or video capturing systems are expected to operate not only in presence of appreciable light but also in low light, especially during the night times. With the application extending to such cases, the low light images that are captured by the systems are not clear enough to serve the purpose of what they are supposed to serve. The images which are captured in low light are susceptible to a decrease in contrast as well as a reduction in its resolution. The scene details may also appear to be blurred with a reduction in the image resolution. This creates a serious concern especially when the image capturing or video capturing systems are being used for security surveillance. The application areas where low light images are usually appearing include medical imaging, which again is a critical application where the image resolution is of prime importance. Since the application areas involve such serious use cases, low light image enhancement finds its application more as a necessity rather than a mere feature.

The low light images with reduced contrast and resolution become one of the major concerns in the applications which uses them. Thus resolution enhancement of low light images coupled with contrast enhancement will result in providing a reasonable solution to the problem at hand. Image enhancement using a combination of Iterative back projection and Curvelet transforms is proposed in this research work for resolution and contrast enhancement. Iterative back projection is a principal concept in image reconstruction. This concept can be visualized as a sketch artist who sketches a portrait of a person with the help of certain key features. Even though the artist may not be able to completely replicate the exact portrait, however, the artist may be successful in drawing a reasonably good portrait by drawing the features explained. The more the number of features, the closer will be the drawn product to the actual requirement. Several enhancements have been made to this fundamental concept of iterative back projection, but still, it has its use in image reconstruction. The rest of the paper is organized as follows: Section “Literature review” gives the brief about the research articles referred, Section “Methodology” is the description of the proposed research work, Section “Simulation results” is results and comparison using performance metrics and Section “Conclusion” is the conclusion and scope for future work.

## Literature review

Low light image restoration or enhancement is a technique where a digital image is improved upon its quality so that it becomes feasible to use them for applications where quality plays a crucial role. A considerable amount of literature related to image enhancement has been reviewed. Through this review, it was understood that the enhancement algorithms are classified into those working in the spatial domain and those in the transform domain. The enhancement can also be performed using several filtering techniques. The transform domain techniques are again classified into wavelet domain and curvelet domain algorithms based on the domain in which they work. The reviewed papers include resolution enhancement with iterative back projection^1–4^, the papers which explain the concept of Gaussian Mixture Model(GMM) and curvelet transform^5–7^.

## Methodology

**Figure 1:**
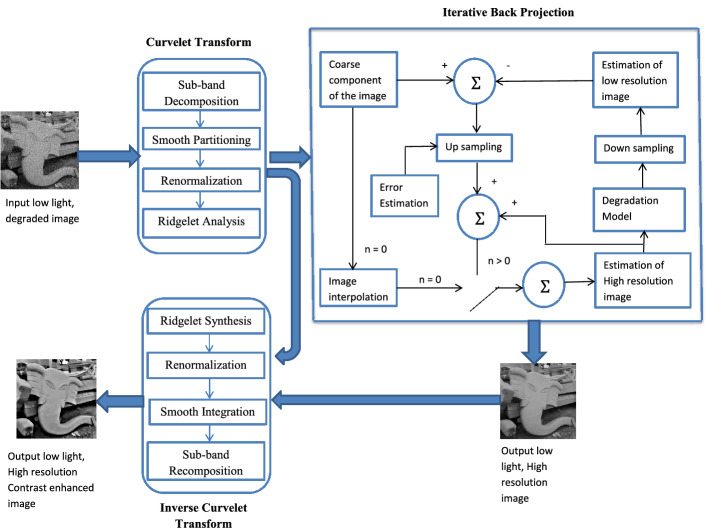
The complete process of image resolution enhancement.

In this section, the method proposed in this work is presented. The block diagram of the entire process is shown in the Fig. 1. In the proposed method, a low light, low resolution image is taken as the input image. Input is then transformed to the curvelet domain. Curvelet transform gives good sparsity for images with curves and edges. The low light images, in general, contain a lot of noise, and in order to remove the noise a pre-filtering stage is applied. This pre-filtering reduces the image quality in terms of resolution and in order to improve the image resolution, an iterative back projection algorithm with gradient descent optimization is used here. Usage of this modified method which uses iterative back projection with curvelet transform not only improves the resolution of the image but also retains the edge details from the image. Iterative back projection (IBP) is a process that initially performs an approximate guess and progresses iteratively to minimize the error that is observed between the estimated image and the input image. Once the error is minimized to an acceptable level, the iterations are stopped. The output obtained at this stage is a resolution enhanced image. This, in turn, passes through the inverse curvelet stage and the final output will be an enhanced quality image both in terms of contrast and resolution.

The details of the different stages involved in the process are given below:

- *Stage 1* This stage is a curvelet decomposition stage, where the image is decomposed into different resolution layers, The details of the steps involved in curvelet transform implementation are given below:
  - Sub-band Decomposition
    It divides the input into different frequency layers. This operation is more similar to a convolution operation. Decomposed layers are given as
    1 $$\begin{aligned} I\mapsto (P_0I, \Delta _1I, \Delta _2I, \ldots) \end{aligned}$$
    where *I* is the input to be decomposed, $P_0$ is the low frequency layer and $\Delta _1, \Delta _2, \ldots$ are the high frequency layers.
  - Smooth Partitioning
    It performs a scaling operation where each frequency layer is divided into squares of appropriate scale. A grid of dyadic squares, *Q* is defined for this purpose. It is represented as
    2 $$\begin{aligned} \Delta _sI\mapsto (w_Q\Delta _sI)_{Q\in \mathcal {Q}_s} \end{aligned}$$
    $\mathcal {Q}$ represents the group of all *Q* and $\mathcal {Q}_s$ is the collection of dyadic squares of scale *s*. *w* is the windowing function used for smooth partitioning and displacement of window localized near *Q* is represented as $w_Q$. Dividing the sub-band into squares is done by multiplying $\Delta _sI$ with $w_Q$.
  - Renormalization
    It is the process of normalizing the dyadic squares into unit squares. For each *Q* the transport operator $S_Q$ on *I* is defined as
    3 $$\begin{aligned} (S_QF)(x_1,x_2)=2^sf(2^sx_1-k_1,2^sx_2-k_2) \end{aligned}$$
    and normalizing the squares are done as
    4 $$\begin{aligned} g_Q=(S_Q)^{-1}(w_q\Delta _sI), \quad \quad Q\in \mathcal {Q} \end{aligned}$$
  - Ridgelet Analysis
    Ridgelet transform is used to analyze the normalized squares. In this step, the ridgelet transform is applied to the combined dyadic squares.
- *Stage 2* This stage is a pre-filtering process and this pre-filtering technique uses a Gaussian Mixture Model based denoising process^5^.
- *Stage 3* This stage consists of the application of an iterative back projection algorithm to the coarse component of the transformed image. Here the IBP algorithm uses a gradient descent optimization technique which helps in achieving high quality solutions. The optimization technique uses a constant step function in order to optimize the selection of HR images to obtain the estimation of the same. The main objective of IBP is to minimize the error between the simulated and observed data. The steps are given as follows:
  - *Step 1:*Make the initial guess of the high resolution image by decimating the low resolution input image.
  - *Step 2:*Using this high resolution image simulate a low resolution version of it with the help of a down sampling operation.
    5 $$\begin{aligned} I_{CLR}^{(n)} = (I_{CHR}^{(n)})\downarrow d \times B \end{aligned}$$
    where $I_{CLR}$ is the coarse component of the low resolution image, $I_{CHR}$ is the coarse component of the high resolution image, $\downarrow d$ is the decimation operator and *B* is the degradation function. (*n*) represents $n^{th}$ iteration.
  - *Step 3:*Estimate the HR image by back propagating the difference between the input low resolution image and simulated low resolution image.
    6 $$\begin{aligned} I_{CHR}^{(n+1)} = I_{CHR}^{(n)} + \Sigma ({I_{CLR} -I_{CLR}^{(n)}}) \times H^{BP} \end{aligned}$$
    $H^{BP}$ is the back projection kernel.
  - *Step 4:*Check the error, which is the difference between the input low resolution image and simulated low resolution image.
    7 $$\begin{aligned} error = I_{CLR} -I_{CLR}^{(n)} \end{aligned}$$
  - *Step 5:*Continue steps 2, 3 and 4 until the energy of the error is minimum.The output of this stage is high resolutiontion coarse component.

- *Step 4 *This is the final stage in the enhancement process. In this stage, the inverse curvelet transform of the resolution enhanced coarse component is executed. The different steps involved in this stage are Ridgelet Synthesis, Renormalization, Smooth integration, and Sub-band recomposition. This is in essence is the reverse of the process detailed in Stage 1. The final output after the inverse curvelet transform is an image with enhanced quality both in terms of resolution and contrast.

The analysis of the quality of the enhanced image is done with the help of the quality metrics, Peak Signal to Noise Ratio(PSNR) and Structural Similarity Index(SSIM)^8^.

## Simulation results

Simulations were done in MATLAB2018a and a set of natural low light images were used to analyse the performance of the proposed method. A database of low light images was built for experimental purposes. This database which is known as ‘Dimair’^9^ has approximately 300 images acquired in a low light environment and used as input to the proposed research work. The resolution enhancement results from the proposed method were compared with the results of resolution enhancement obtained using conventional iterative back projection algorithm^2^ and resolution enhancement method which uses Edge Preserving IBP^3^ .

Images of Car, Sculpture, Building, and Sky from the ’Dimair’ data set are taken for the simulation.

**Figure 2:**
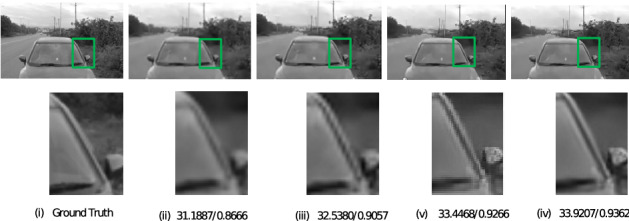
Result of enhancement on Car image with PSNR and SSIM values (i) Ground Truth (ii) Degraded low resolution Image (iii) Result of conventional IBP enhancement technique (iv) Result of Edge preserving IBP (v) Result of the proposed method.

**Figure 3:**
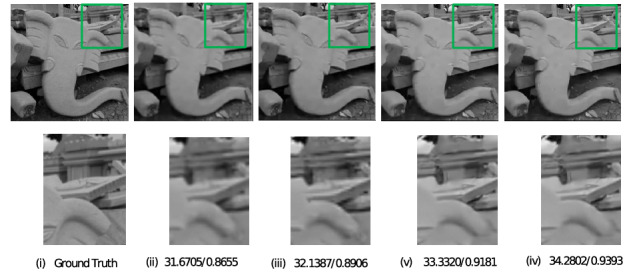
Result of enhancement on Sculpture image with PSNR and SSIM values (i) Ground Truth (ii) Degraded low resolution Image (iii) Result of conventional IBP enhancement technique (iv) Result of Edge preserving IBP (v) Result of the proposed method.

**Figure 4:**
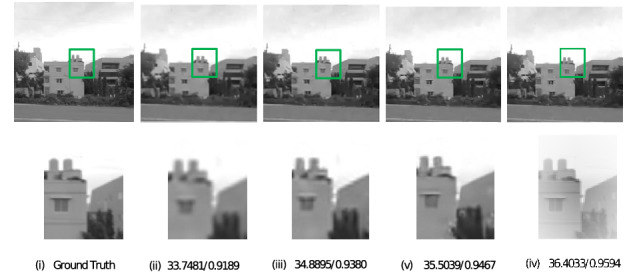
Result of enhancement on Building image with PSNR and SSIM values (i) Ground Truth (ii) Degraded low resolution Image (iii) Result of conventional IBP enhancement technique (iv) Result of Edge preserving IBP (v) Result of the proposed method.

**Figure 5:**
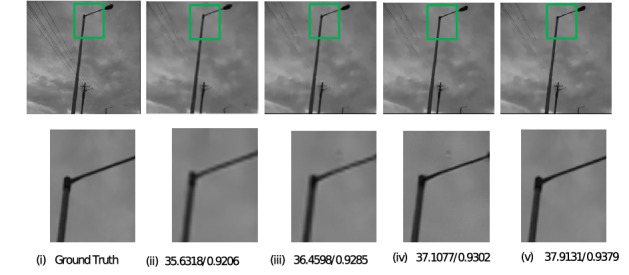
Result of enhancement on Sky image with PSNR and SSIM values (i) Ground Truth (ii) Degraded low resolution Image (iii) Result of conventional IBP enhancement technique (iv) Result of Edge preserving IBP (v) Result of the proposed method.

The images selected for simulation were all captured in low light environment and the intensity of light was measured using a lux meter. These meter readings were taken at the instants when the images were captured. The simulation was performed on images of car which are taken at light intensities 232,262,282,315, images of sculpture captured at light intensities 84,112,140,152, images of the building taken at light intensities 230,253,298,321, and images of sky captured at light intensities 270,287,302,330 (unit is lux). For all these images, during a pre-processing stage^9^, the image quality was reduced which resulted in deterioration of resolution. The proposed method was applied to this low resolution images along with conventional iterative back projection resolution enhancement algorithm^2^ and resolution enhancement method which uses Edge Preserving IBP^3^. Figures 2, 3, 4, and 5 show the result of the three methods for a single intensity image of Car, Sculpture, Building and Sky.

The figures show the result for the Car image at the intensity of 315 lux, the Building image at an intensity of 321 lux, the Sculpture image at an intensity of 152, and the Sky image at the intensity of 330 lux. From the results shown, it can be observed that the proposed method gives better visual quality for images compared to the conventional iterative back projection method and resolution enhancement method which uses Edge Preserving IBP.

For all the images at different intensity levels, PSNR and SSIM values were calculated and noted. In the above figures, PSNR and SSIM values for that particular intensity level are mentioned below each image. It is seen that the proposed method is better in terms of both PSNR and SSIM.

Also the PSNR and SSIM values are tabulated for the images with different intensity levels and are shown in Tables 1 and 2. It is clear from the tables that the proposed method gives better results in terms of PSNR and SSIM values compared to the conventional IBP algorithm and resolution enhancement method which uses Edge Preserving IBP. The tabulated PSNR and SSIM values for different images are plotted against the intensity level of the images.

**Table 1:** Peak Signal to Noise Ratio(PSNR) in dB.

Image	Light intensity (lux)	LR image	Simple IBP^2^	Edge preserving IBP^3^	Proposed method
Car	315	31.1887	32.5380	33.4468	33.9207
	282	31.2314	32.4676	33.1268	33.8358
	261	31.4316	32.8427	33.8826	34.1294
	232	31.8876	32.9931	33.5839	34.4800
Sculpture	152	31.6705	32.1387	33.3320	34.2802
	140	31.6135	32.0456	33.4517	34.2416
	112	31.7158	32.0020	33.2973	34.1856
	84	31.9885	32.2894	33.4425	34.5202
Building	321	33.7481	34.8895	35.5039	36.4033
	298	33.6530	34.7269	35.3755	36.3275
	253	33.8404	34.6821	35.2974	36.3292
	230	34.4413	34.9918	35.5183	37.0707
Sky	330	35.6318	36.4598	37.1017	37.9131
	302	35.4936	36.2601	36.9852	37.7564
	287	35.7232	36.5108	37.0097	37.8153
	270	35.4576	36.1173	37.5931	38.1801

**Table 2:** Structural Similarity Index(SSIM).

Image	Light intensity (lux)	LR image	Simple IBP^2^	Edge preserving IBP^3^	Proposed method
Car	315	0.8666	0.9057	0.9266	0.9362
	282	0.8669	0.9028	0.9249	0.9327
	261	0.8607	0.9012	0.9237	0.9306
	232	0.8602	0.8994	0.9191	0.9290
Sculpture	152	0.8655	0.8906	0.9181	0.9393
	140	0.8574	0.8865	0.9195	0.9353
	112	0.8575	0.8806	0.9146	0.9345
	84	0.8590	0.8893	0.9177	0.9359
Building	321	0.9189	0.9380	0.9467	0.9594
	298	0.9147	0.9362	0.9482	0.9555
	253	0.9140	0.9358	0.9469	0.9558
	230	0.9134	0.9341	0.9438	0.9549
Sky	330	0.9233	0.9285	0.9302	0.9379
	302	0.9192	0.9227	0.9286	0.9339
	287	0.9168	0.9214	0.9293	0.9312
	270	0.9199	0.9223	0.9299	0.9326

**Figure 6:**
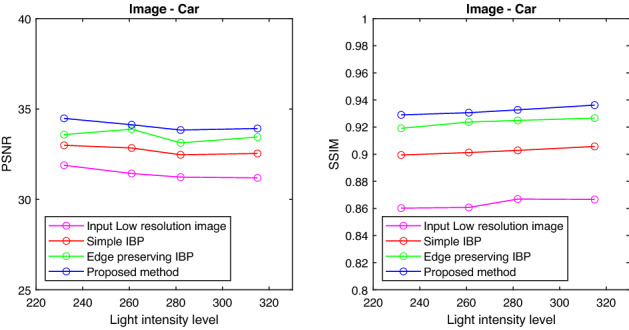
PSNR and SSIM plotted against light intensities—Car image.

**Figure 7:**
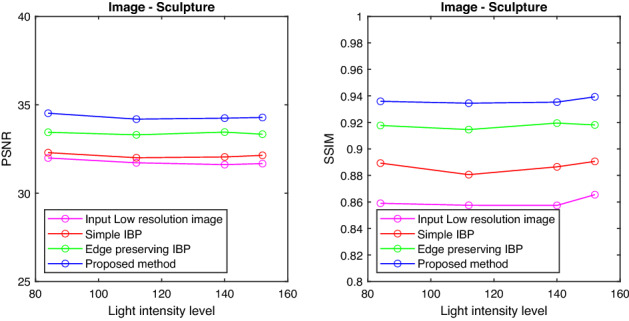
PSNR and SSIM plotted against light intensities—Sculpture image.

**Figure 8:**
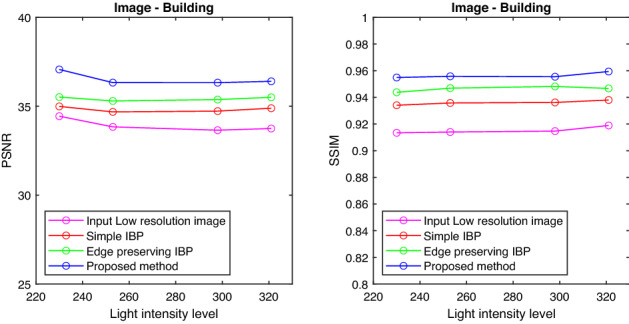
PSNR and SSIM plotted against light intensities—Building image.

**Figure 9:**
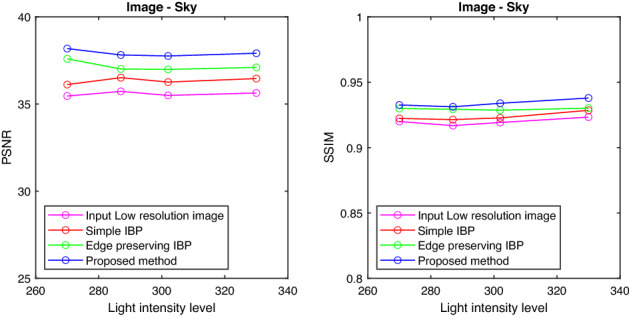
PSNR and SSIM plotted against light intensities—Sky image.

Figures 6, 7, 8, and 9 show the graph of PSNR and SSIM plotted against light intensities for Car, Sculpture, Building and Sky images. From the graph, we can see that the proposed method is efficient in enhancing the resolution of degraded images at different intensity levels.

**Figure 10:**
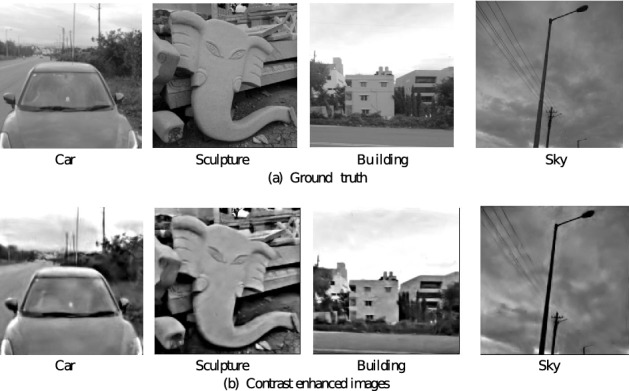
Result of contrast enhancement on low light images.

**Figure 11:**
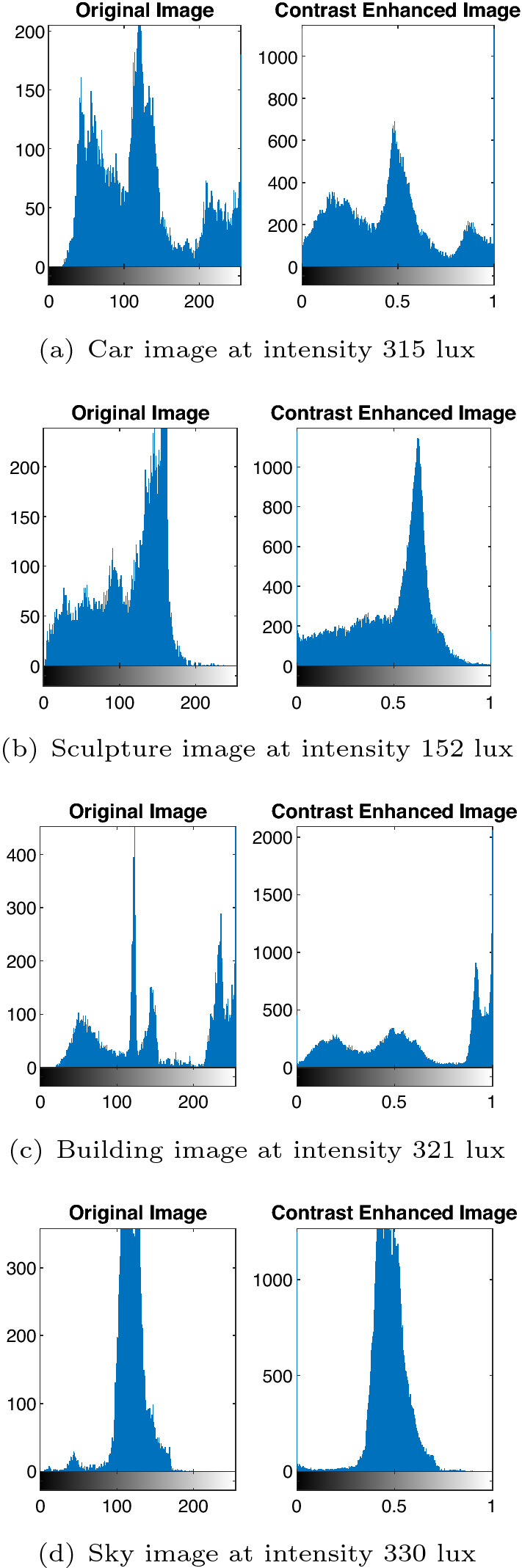
Histogram for the ground truth and contrast enhanced images.

The result of contrast enhancement on the low light images for one intensity level is given in Fig. 10 along with the original images. The Car image is at an intensity level of 315 lux, the Sculpture image at 152 lux, the Building image at 321 lux, and the Sky image at 330 lux. The histogram plot for these original images and the contrast enhanced images are shown in Fig. 11. The difference in contrast between the original image and contrast enhanced image is clearly visible from the plotted histogram.

In order to check the improvement in terms of contrast, we have calculated Absolute mean brightness difference and entropy for the ground truth and contrast enhanced image from the method which combines curvelet and IBP. The values of mean brightness and absolute brightness difference is recorded in the Table 3. The values of entropy for the captured image and contrast enhanced image is tabulated in the Table 4. From Table 3, it is seen that the mean brightness of the contrast enhanced output image is higher than the mean brightness of the input ground truth images taken for simulation. This clearly indicate there is an improvement in the contrast of the output image. The value of difference in brightness is also recorded in the same table. From the entropy values of the input ground truth and entropy values of the enhanced image given in Table 4, we can see that the entropy of the enhanced image is higher than the entropy of the input image. This shows the efficiency of the proposed algorithm in retaining the information content.

**Table 3:** Mean Brightness and Absolute Mean Brightness Difference.

Image	Light intensity (lux)	Mean brightness (Ground truth)	Mean brightness (Contrast Enhanced)	Absolute mean brightness difference
Car	315	119.1836	121.3954	2.2118
	282	97.6295	99.4904	1.8609
	261	83.1039	85.8261	2.7221
	232	69.7395	73.7576	4.0181
Sculpture	152	103.5094	106.1310	2.6216
	140	96.1556	99.2778	3.1221
	112	88.5400	92.3828	3.8427
	84	74.8688	78.9262	4.0574
Building	321	165.3693	167.0435	1.6741
	298	150.9501	151.0648	0.1147
	253	128.2551	129.4830	1.2280
	230	109.4688	111.5200	2.0512
Sky	330	117.7276	118.2393	0.5117
	302	98.8083	99.7315	0.9232
	287	79.0154	80.1068	1.0914
	270	56.7000	57.6497	0.9498

**Table 4:** Entropy of the images.

Image	Light intensity (lux)	Entropy–ground truth (dB)	Entropy–enhanced image (dB)
Car	315	7.4610	7.5270
	282	7.2756	7.5244
	261	7.0194	7.4300
	232	6.7115	7.2108
Building	321	6.6685	6.8985
	298	6.7061	7.4007
	253	6.6425	7.1743
	230	6.3291	6.7566
Sculpture	152	7.1148	7.1689
	140	6.9821	7.0919
	112	6.8534	6.9973
	84	6.5521	6.7580
Sky	330	6.0670	6.3758
	302	6.0574	6.3147
	287	6.0333	6.2919
	270	5.9573	6.2079

## Conclusion

An image enhancement method that combines curvelet transform and iterative back projection is implemented in this paper. The quality of the output image is improved in terms of both resolution and contrast. A database ‘Dimair’ has been built to store low light images and the experiment was done by taking a few of these images as the input. The traditional iterative back projection and the method which uses edge preserving IBP are compared with the method that combines curvelet transform with iterative back projection, in terms of PSNR and SSIM, and the results suggest affirmative for the proposed method. The proposed method has been found to perform better not only with the enhancement of resolution but also with the performance in terms of edge enhancement with reduced ringing artefacts. Here the primary aim was to improve the image resolution and the work can be further extended by introducing techniques to have more improvements in contrast enhancement of colour images acquired in the low light environment.

## Data Availability

All data generated or analysed during this study are included in this published article. Also they are available from the corresponding author upon request.
